# Paradox of Vaccination: Is Vaccination Really Effective against Avian Flu Epidemics?

**DOI:** 10.1371/journal.pone.0004915

**Published:** 2009-03-18

**Authors:** Shingo Iwami, Takafumi Suzuki, Yasuhiro Takeuchi

**Affiliations:** 1 Graduate School of Science and Technology, Shizuoka University, Shizuoka, Japan; 2 Graduate School of Engineering, Shizuoka University, Shizuoka, Japan; University of Maryland, United States of America

## Abstract

**Background:**

Although vaccination can be a useful tool for control of avian influenza epidemics, it might engender emergence of a vaccine-resistant strain. Field and experimental studies show that some avian influenza strains acquire resistance ability against vaccination. We investigated, in the context of the emergence of a vaccine-resistant strain, whether a vaccination program can prevent the spread of infectious disease. We also investigated how losses from immunization by vaccination imposed by the resistant strain affect the spread of the disease.

**Methods and Findings:**

We designed and analyzed a deterministic compartment model illustrating transmission of vaccine-sensitive and vaccine-resistant strains during a vaccination program. We investigated how the loss of protection effectiveness impacts the program. Results show that a vaccination to prevent the spread of disease can instead spread the disease when the resistant strain is less virulent than the sensitive strain. If the loss is high, the program does not prevent the spread of the resistant strain despite a large prevalence rate of the program. The epidemic's final size can be larger than that before the vaccination program. We propose how to use poor vaccines, which have a large loss, to maximize program effects and describe various program risks, which can be estimated using available epidemiological data.

**Conclusions:**

We presented clear and simple concepts to elucidate vaccination program guidelines to avoid negative program effects. Using our theory, monitoring the virulence of the resistant strain and investigating the loss caused by the resistant strain better development of vaccination strategies is possible.

## Introduction

Highly pathogenic H5N1 influenza A viruses have spread relentlessly across the globe since 2003. They are associated with widespread death of poultry, substantial economic loss to farmers, and reported infections of more than 300 people with a mortality rate of 60% [1]. Influenza prevention and containment strategies can be considered under the broad categories of antiviral, vaccine, and non-pharmaceutical measures [2], [3], [4], [5], [6], [7], [8], [9], [10], [11], [12], [13]. A major public health concern is the next influenza pandemic; yet it remains unclear how to control such a crisis.

Vaccination of domestic poultry against the H5N1 subtype of avian influenza has been used in several countries such as Pakistan, Hong Kong, Indonesia, China, and Vietnam [14], [15], [16]. Using vaccination to reduce the transmission rate might provide an alternative to mass culling, by reducing both the susceptibility of healthy birds and the infectiousness of infected birds [14], [17], [18]. However, incomplete protection at the bird level can cause the silent spread of the virus within and among birds [11]. Furthermore, vaccines might provide immunological pressure on the circulating strains, which might engender the emergence of drifted or shifted variants with enhanced potential for pathogenicity in humans [1]. Therefore, although vaccination programs have been recommended recently, some field evidence indicates that vaccination alone will not achieve eradication. Moreover, if not used appropriately, vaccination might result in the infection becoming endemic [11], [17].

An important issue related to influenza epidemics is the potential for the emergence of vaccine-resistant influenza viruses. The vaccine-resistant strain, in general, causes a loss of the protection effectiveness of vaccination [19], [20], [21], [22] (there is experimental evidence of the loss of the protection effectiveness for antiviral-resistant strains [23]). Consequently, a vaccination program that engenders the emergence of the resistant strain might promote the spread of the resistant strain and undermine the control of the infectious disease, even if the vaccination protects against the transmission of a vaccine-sensitive strain [20], [21], [22].

For example, in China, despite a compulsory program for the vaccination of all poultry commencing in September 2005, the H5N1 influenza virus has caused outbreaks in poultry in 12 provinces from October 2005 to August 2006 [14], [15], [22]. Genetic analysis revealed that an H5N1 influenza variant (Fujian-like, FJ like), which is a previously uncharacterized H5N1 virus sublineage, had emerged and subsequently became the prevalent variant in each of the provinces, replacing those previously established multiple sublineages in different regions of southern China. Some data suggest that the poultry vaccine currently used in China might only generate very low neutralizing antibodies to FJ-like viruses (seroconversion rates remain low and vaccinated birds are poorly immunized against FJ-like viruses) in comparison to other previously cocirculating H5N1 sublineages [20], [22]. That evidence implies the possibility that the emergence and replacement of FJ-like virus was preceded by and facilitated by the vaccination program, although the mechanism remains unknown epidemiologically and virologically (some researchers consider that the emergence and replacement of FJ-like virus are questionable [24], [25]).

Furthermore, the H5N2 vaccines have been used in Mexico since 1995 [17], [19], [21]. Phylogenetic analysis suggests the presence of (previously uncharacterized) multiple sublineages of Mexican lineage isolates which emerged after the introduction of the vaccine. Vaccine protection studies further confirmed in vitro serologic results indicating that commercial vaccine was not able to prevent virus shedding when chickens were challenged with the multiple sublineage isolates [19], [21]. Therefore, the vaccine protective efficacy would be impaired and the use of this specific vaccine would eventually become obsolete. That fact also implies that the vaccine promotes the selection of mutation in the circulating virus.

The emergence of a vaccine-resistant strain presents the risk of generating a new pandemic virus that is dangerous for humans through an avian-human link because of the spread of vaccine-resistant strain. The dynamics of competition between vaccine-sensitive and vaccine-resistant strains is, in general, complex [8], [9]. Actually, outcomes of the dynamics might be influenced by several factors, including a loss of protection effectiveness, a competitive advantage of vaccine-resistant strain, and a prevalence rate of vaccination. Understanding the dynamics of a spread of vaccine-resistant is therefore crucial for implementation of effective mitigation strategies.

Several theoretical studies have investigated the impact of an emergence of a resistant strain of antiviral drug such as M2 inhibitors and NA inhibitors during an influenza pandemic among humans [2], [3], [8], [9], [10], [12], [26]. However, to our knowledge, no study has used a mathematical model to investigate the application of vaccination program among poultry in the context of an emergence of a vaccine-resistant strain. It remains unclear whether a vaccination program can prevent the spread of infectious disease when the vaccine-resistant strain emerges and how a loss of immunization by vaccination within birds infected with the vaccine-resistant strain affects the spread of infectious disease among birds. Nobody can give a simple and clear explanation to capture the problems described above in a theoretical framework (using numerical simulations, many qualitative and quantitative but sometimes very complex studies have investigated effects of antiviral drugs [3], [8], [9], [10], [12], [26]). Furthermore, we remain skeptical that a vaccination program can reduce the number of total infectious individuals even if the vaccination protects against transmission of a vaccine-sensitive strain. We developed a simple mathematical model to evaluate the effectiveness, as a strategy to control influenza epidemic, of a vaccination program among poultry which can engender the emergence of a vaccine-resistant strain.

## Methods

**Figure 1:**
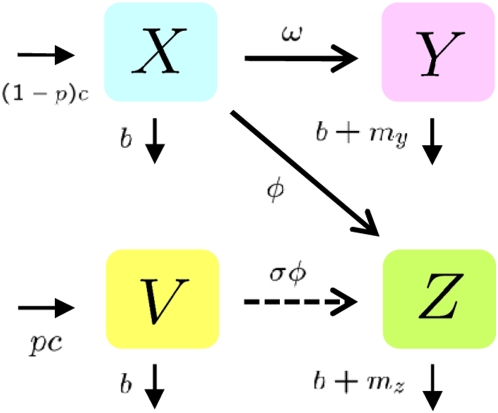
Model structure for the emergence of vaccine-resistant strain during a vaccination program: Susceptible birds (*X*) become infected with vaccine-sensitive (*Y*) and vaccine-resistant (*Z*) strains at rates in direct relation to the number of respective infectious birds. We assume that vaccinated birds (*V*) can be protected completely from the vaccine-sensitive strain, but are partially protected from vaccine-resistant strains with a loss of protection effectiveness of the vaccination (*σ*). See the Mathematical model section for corresponding equations.

Herein, we describe a homogeneous population model of infectious disease and its control using a vaccination program in the presence of a vaccine-resistant strain (Fig. 1).

All birds in the effective population are divided into several compartments, respectively including susceptible birds (*X*), vaccinated birds (*V*), birds infected with vaccine-sensitive strain (*Y*), and birds infected with vaccine-resistant strain (*Z*). We assume that susceptible birds are born or restocked at a rate of *c* per day and that all birds are naturally dead or removed from the effective population at a rate of *b* per day.

In the absence of vaccination, transmission occurs at a rate that is directly related to the number of infectious birds, with respective transmission rate constants *ω* and *φ* from infected birds with the vaccine-sensitive strain and with the vaccine-resistant strain. The infectiousness of vaccine-sensitive and vaccine-resistant strain are assumed to be exponentially distributed, respectively, with mean durations of 1/(*b*+*my*) and 1/(*b*+*mz*) days. Actually, *my* and *mz* respectively signify virulence of vaccine-sensitive and vaccine-resistant strains.

At the beginning of the vaccination program, *X* moves directly to *V* by the vaccination. However, after some period after the initial vaccination, the direct movement might vanish because almost all birds are vaccinated. Therefore, we can assume that vaccination is only administered to the newly hatched birds. The newly hatched birds are vaccinated at the rate 0≤*p*≤1 (more appropriately, *p* is proportional). Actually, *p* represents the prevalence rate of the vaccination program.

To simplify the theoretical treatment, as described in [11], we assume that the vaccinated birds can be protected completely from the vaccine-sensitive strain (note that the assumption is not necessary for our results: see Supplementary Information: Text S1, Fig. S10, S11). Actually, in laboratory experience, many avian influenza vaccines confer a very high level of protection against clinical signs and mortality (90–100% protected birds) [21]. However, many factors determine whether a vaccinated bird becomes infected, including age, species, challenge dose, health, antibody titre, infections of immunosuppressive diseases, and cross-reactivity of other avian influenza serotypes [11], [27], [28], [29]. On the other hand, we assume that the vaccinated birds are partially protected from the vaccine-resistant strain at the rate (proportion) 0≤1−*σ*≤1 because of cross-reactivity of immune systems [19], [20], [22], [23], [29] (e.g., *σ* = 0 represents complete cross immunity against vaccine-resistant strains). Actually, *σ* represents a loss of protection effectiveness of the vaccination caused by a vaccine-resistant strain.

### Mathematical model

We extended the standard susceptible–infective model [30] including the effect of a vaccination program that can engender the emergence of a vaccine-resistant strain. Our mathematical model is given by the following equations:
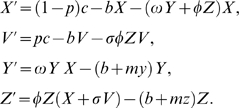
(1)Model (1) is a simplified one that is used in [31]. We considered a mechanism for the emergence and replacement of the FJ-like virus over a large geographical region in China using a more complex patch-structured model in the heterogeneous area [31]. Here we investigate the impact of the vaccination program in a homogeneous area and specifically examine the role of epidemiological parameters such as the prevalence rate of the vaccination program (*p*) and the loss of protection effectiveness of the vaccination (*σ*) in the spread of the disease.

### Estimation of epidemiological parameters

Baseline values of model parameters and their respective ranges used for simulations are presented in Table 1 and 2. These parameters are based on avian influenza epidemics among poultry in The Netherlands in 2003 [32], [33], [34].

**Table 1:** Description of physical characteristics, transmission, infectious, and vaccination parameters of the model with their baseline values and ranges used for simulations.

Symbol	Description	Value (Range)	Reference
*c/b*	Initial bird population size	984 birds	[34]
1/(*b*+*my*)	Mean infectious period of V-S strain	13.8 days	[34], [36]
*ω*	Transmissibility of V-S strain	4.78×10^−4^ day^−1^ individual^−1^	[34]
(*b*+*my*)/(*b*+*mz*)	Relative mean infectious period of V-R strain	1.32	[10], [12], [23]
*ϕ/ω*	Relative transmissibility of V-R strain	0.58	[10], [12], [23]
*σ*	Loss of vaccine effectiveness by V-R strain	Variable (0–1)	–
*p*	Prevalence rate of vaccination program	Variable (0–1)	–

These parameters are based on avian influenza epidemics in The Netherlands in 2003 [32], [33], [34]. Actually, V-S and V-R represent “vaccine-sensitive” and “vaccine-resistant”, respectively.

**Table 2:** Basic reproductive numbers and invasion reproductive numbers before the vaccination program.

Symbol	Description	Value	Reference
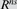	Basic reproductive numbers of vaccine-sensitive strain	6.53	[34], [36]
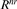	Basic reproductive number of vaccine-resistant strain	4.99	[10], [12], [23]
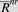	Invasion reproductive number of vaccine-resistant strain	0.76	–

These values are based on the H7N7 epidemic in The Netherlands in 2003 [32], [33], [34]. The exact expressions of these reproductive numbers are given in Supplementary Information: Text S1.

The initial population size was *c/b* = 984 birds at the 2003 epidemic [34]. Usually, the mean lifespan of poultry is about 2 years. However, we assume that the mean duration of a bird being in effective population is about 1/*b* = 100 days because of migration and marketing. Therefore, the birth or restocking rate of birds is *c* = 9.84 birds per day. Estimated infectious period and transmission parameters are 1/(*b*+*my*) = 13.8 days and *ω* = 4.78×10^−4^ day^−1^ individual^−1^, respectively, [34]. These physical characteristics, in addition to infectious and transmission parameters, are used in our model as parameters of the vaccine-sensitive strain.

The epidemiological and biological feature of antiviral drug-resistance is well reported in [23]. The transmissibility and virulence of drug-resistant strains are usually lower than those of the wild strain because of its mutation cost [8], [10], [23], [35]. Actually, antiviral drugs are also used for prophylaxis drug intervention as vaccination [8], [10], [12]. Herein, we use some reduced value of transmissibility (*φ/ω* = 0.58) and the increased value of infectious period of the vaccine-sensitive strain ((*b*+*my*)/(*b*+*mz*) = 1.32) for parameters of vaccine-resistant strain (sensitivity analyses are given in Supplementary Information: Text S1, Fig. S6, S7, S8, S9).

### Reproductive numbers

A measure of transmissibility and of the stringency of control policies necessary to stop an epidemic is the basic reproductive number, which is the number of secondary cases produced by each primary case [30]. We obtain basic reproductive quantities of vaccine-sensitive strain 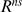 and vaccine-resistant strain 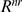 before vaccination program (superscript *n* means no vaccination). In fact, during the vaccination program, the basic reproductive numbers depend on the rate of prevalence of the vaccination program. We derived these basic reproductive numbers depending on the prevalence rate in Supplementary Information: Text S1. With the estimated parameters in Table 1 the basic reproductive number of vaccine-sensitive and vaccine-resistant strain are 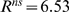 and 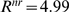, respectively (note that 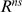 corresponds to an estimated value in [34]).

Furthermore, to clarify the concept of competition among strains simply, we introduce the invasion reproductive number for the vaccine-resistant strain before the vaccination program 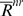, which signifies an expected number of new infectious cases with the vaccine-resistant strain after a spread of a vaccine-sensitive strain among birds. The invasion reproductive number is considered as a competitive condition (relative fitness), which represents some advantage measure of the vaccine-resistant strain against the vaccine-sensitive strain. The estimated invasion reproductive number of the vaccine-resistant strain is 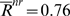. During the vaccination program, the invasion reproductive number also depends on the prevalence rate of the vaccination program (see Supplementary Information: Text S1).

## Results

We consider a scenario in which a vaccine-resistant strain can emerge (i.e., be eventually selected) during a vaccination program designed to be effective against the spread of a vaccine-sensitive strain. This implies that 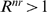: otherwise the vaccine-resistant strain can not emerge at all (see Supplementary Information: Text S1, Fig. S1, S2, S3). Acquisition of resistance ability usually engenders a strain which, in the absence of a pharmaceutical intervention, is less fit than the sensitive strain [8], [9], [12], [35]. Therefore, 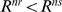. We generally assume the following conditions for reproductive numbers before the vaccination program (our baseline parameter values are satisfied with these assumptions):
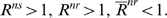



The assumption precludes the possibility that a pre-existing vaccine-resistant strain beats the vaccine-sensitive strain before the vaccination program because 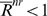.

### Evaluation of the effect of a vaccination program

**Figure 2:**
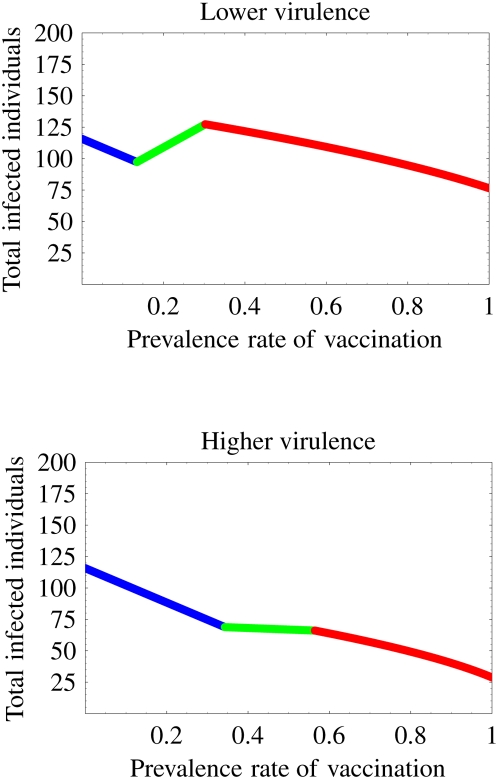
Final size of epidemics related with the prevalence rate of the vaccination: The top figure represents that the vaccination is not always effective in the case of lower virulence of vaccine-resistant strain. The bottom figure represents that the vaccination is always effective in the case of higher virulence of the vaccine-resistant strain. We assume that *σ* = 0.35, *mz* = 0.045 (top) and *mz* = 0.065 (bottom). These values of *σ* and *mz* are not so influential on the result. The blue, green, and red lines respectively signify situations in which only the vaccine-sensitive strain exists, both the vaccine-sensitive and the vaccine-resistant strains exist, and only the vaccine-resistant strain exists.

Although vaccination is an important tool to control epidemics, the use of vaccination might engender a spread of a vaccine-resistant strain. To demonstrate the interplay between these opposing effects, we simulated our model to determine the final size of an epidemic (total infected individuals *Y*+*Z* at equilibrium level) over vaccination prevalence (0≤*p*≤1) in Fig. 2 (we use our baseline parameter values except for *mz*). We assume that the loss of the protection effectiveness is 35% (*σ* = 0.35: this value can be chosen arbitrarily with little effect on the meaning of the results). The estimated infectious period of the vaccine-sensitive strain is 13.8 days [34] (see Table 1). Therefore, the virulence of vaccine-sensitive strain is *my* = 0.062 day^−1^. Results show that the patterns of the final size can be divided into two cases, which depend strongly on the virulence of the vaccine-resistant strain. If the virulence of the vaccine-resistant strain is lower than that of vaccine-sensitive strain (e.g., we choose *mz* = 0.045), then increasing the prevalence rate of vaccination from 13.5% to 30.3% can increase the final size (green line at top figure in Fig. 2). On the other hand, if the virulence is higher (*mz* = 0.065), increasing the prevalence always decreases the final size (bottom figure in Fig. 2). These two patterns are qualitatively preserved for different virulence of the vaccine-resistant strain.

In [8], [9], although they consider the emergence of an antiviral drug-resistant virus, a similar tendency (increasing the treatment level increases the final size of the epidemic) was obtained through complex models that are difficult to treat mathematically. The mathematical model presented herein demonstrates that the patterns of final size over vaccination prevalence only depend on the virulence of the vaccine-resistant strain as follows (see Supplementary Information: Text S1). Increasing the prevalence rate increases the final size when only both strains co-exist if the virulence of vaccine-resistant strain is lower than that of vaccine-sensitive strain (*my*>*mz*). That is to say, the vaccination is effective when either a vaccine-sensitive or a vaccine-resistant strain exists. On the other hand, if the virulence of vaccine-resistant strain is higher than that of vaccine-sensitive strain (*my*<*mz*), the final size always decreases as the prevalence rate increases. The other parameters can not change these patterns. In fact, many studies have ignored the impact of the virulence of the vaccine-resistant strain. In [7], we also found that the virulence of mutant strain determines a choice of the optimal prevention policy for avian influenza epidemic. Therefore, we suggest that, to monitor and investigate the virulence evolution between the vaccine-sensitive and vaccine-resistant strain is important to develop avian flu epidemic plans. In fact, if the vaccine-resistant strain has higher virulence than the vaccine-sensitive strain, the vaccination program is always effective, even though the program engenders the emergence of a vaccine-resistant strain. On the other hand, if the vaccine-resistant strain has lower virulence, we must carefully manage vaccination to prevent the spread of a vaccine-resistant strain.

### Impact of loss of protection effectiveness of vaccination

To ensure an effective vaccination program, the vaccine must protect vaccinated animals against clinical signs of the disease and prevent mortality [21]. However, the vaccine-resistant strain causes a loss of the protection effectiveness of the vaccination [19], [20], [21], [22], [37]. We investigate an impact of the loss of the protection on change of final size of the epidemic over the vaccination prevalence. Assume, hereafter, that the virulence of vaccine-resistant strain is lower than that of vaccine-sensitive strain (*my*>*mz*): otherwise, the vaccination is always effective (our baseline parameter values are satisfied with *my*>*mz*). Actually, a resistant strain seems to have reduced virulence in general [8], [10], [23], [35].

**Figure 3:**
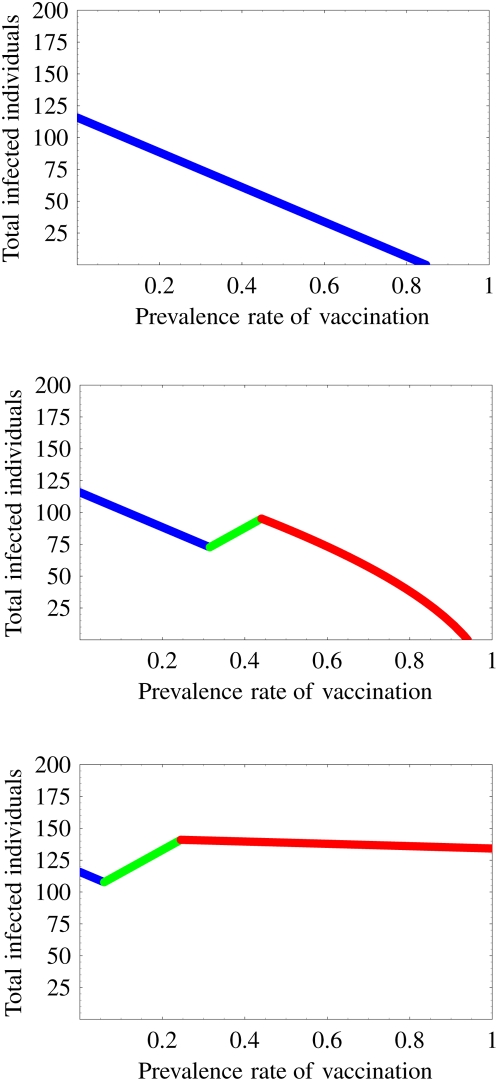
Impact of the loss of the protection effectiveness of the vaccination on the change of the final size of the epidemic: The losses of the protection in the top, middle, and bottom figure are *σ* = 0.05, 0.15, and 0.8, respectively. The top (0≤*σ*≤*σ* ^*^) and middle (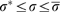) figures portray the possibility of eradication of the infectious disease through the vaccination program. However, in the bottom figure (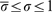), the vaccination engenders a failure to prevent the spread of the disease. The patterns of the change are divisible into these three cases, depending on the loss of the protection. The blue, green, and red lines respectively correspond to the situation in which only the vaccine-sensitive strain exists, both the vaccine-sensitive and the vaccine-resistant strains exist, and only the vaccine-resistant strain exists.

We conduct a simulation using our model to elucidate the change of the final size with the loss of the protection effectiveness 5%, 15%, and 80% over vaccination prevalence in Fig. 3. Results showed that the patterns of the change are divisible into three cases. In theory, we can estimate the threshold values of the loss of the protection which determines the patterns (see Supplementary Information: Text S1, Fig. S4):
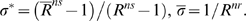



In fact, *σ* ^*^ = 0.056 and 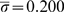 in our simulation from Table 1. When the loss of the protection is between 0% and *σ* ^*^ = 5.6% (5%: the top figure in Fig. 3), the vaccination can control the epidemic with the prevalence rate of 84.7% without the emergence of a resistant strain (a vaccine-resistant strain never emerges in the population). Therefore, increasing the prevalence rate of vaccination always decreases the final size of the epidemic. For the loss of the protection is between *σ* ^*^ = 5.6% and 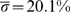 (15%: the middle figure in Fig. 3), the vaccination eventually prevents the spread of the disease with 94.1% of vaccination prevalence in spite of the emergence of the resistant strain. Increasing the prevalence rate from 31.5% to 44.1% increases the final size. Therefore, the vaccination is not always effective. However, when the loss of the protection is between 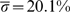 and 100% (80%: the bottom figure in Fig. 3), the vaccination no longer controls the disease (even if the prevalence rate is 100%) and the vaccine-resistant strain spreads widely through the population instead of the vaccine-sensitive strain. In this case, the vaccination only slightly provides beneficial effects for preventing the spread of the disease. Therefore, the loss of the protection effectiveness of vaccination plays an important role in preventing the spread of the disease.

### Vaccination can facilitate spread of disease

Sometimes a considerable spread of the resistant strain partially compromises the benefits of a vaccination program [19], [20], [22], [37]. For example, even if we can completely execute the vaccination program (*p* = 1), the final size of the epidemic can become larger than that before the vaccination program (*p* = 0) by the emergence of vaccine-resistant strain (bottom figure in Fig. 3). This implies that the vaccination, which is expected to prevent the spread of the disease, can instead help the spread of the disease. If the loss of the protection effectiveness of vaccination is high (*σ* ^*^≤*σ*≤1), the vaccination might increase the final size over vaccination prevalence compared with that before the vaccination program (vaccination always decreases the final size if 0≤*σ*≤*σ* ^*^ (top figure in Fig. 3)). Here we can also calculate such a risk of help, which depends on the loss of the protection (see Supplementary Information: Text S1). Let
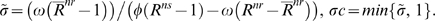



Actually, *σc* = 0.236 in our simulation is from Table 1. When the loss of the protection is between 23.6% and 100%, we found that the vaccination program is attended by the risk that the final size becomes larger than that before the vaccination program (see Supplementary Information: Text S1).

### Difficulty of prediction of a prevalent strain

Vaccination is well known to engender “silent carriers or excretors” if the vaccine can not completely protect the vaccinated animals against clinical signs of the disease [16], [21]. The existence of silent carriers or excretors is dangerous because they become a virus reservoir and shed the virus into their environment, causing potential outbreaks among their own and other species. Furthermore, even if a vaccination is effective in a bird (individual level), an incomplete vaccination program for all birds (population level) can engender the “silent spread” of an infectious disease [1], [11]. Additionally, we found that it is difficult for us to predict a prevalent strain even if we can completely estimate the basic reproductive number of vaccine-sensitive and vaccine-resistant strains during the vaccination program (although estimations, usually, are almost impossible). Even when the basic reproductive number of the vaccine-resistant strain is less than that of the vaccine-sensitive strain (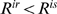), the vaccine-resistant strain can beat the vaccine-sensitive strain and spread widely through the population (see Supplementary Information: Text S1, Fig. S5). Therefore, a non-ideal vaccination program might make a prediction of prevalent strain difficult.

### Optimal prevalence rate of vaccination program

In the absence of a vaccine-resistant strain, a goal of vaccination program is to reduce the basic reproductive number of vaccine-sensitive strain 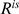 to be less than 1. We assume that 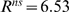. Therefore, the vaccination can eradicate the vaccine-sensitive strain if at least 84.7% of the birds in poultry are vaccinated effectively based on the fraction of 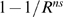 [30]. However, in the presence of the resistant strain, the simple theory is inapplicable to an optimal prevalence rate of vaccination program. Here we define the optimal prevalence rate of a vaccination program which minimizes both the final size of the epidemic and the prevalence rate (see Supplementary Information: Text S1).

**Figure 4:**
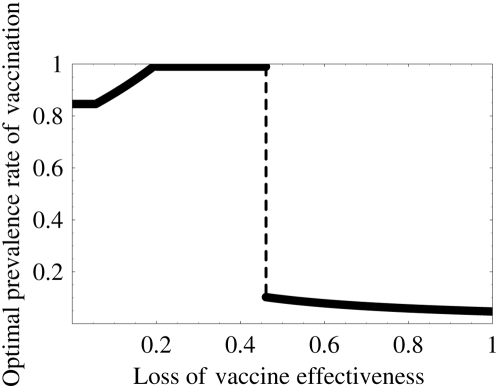
Optimal prevalence rate of vaccination program: Increasing of the loss of the protection effectiveness engenders a catastrophic change in the optimal prevalence rate. The optimal rate increases as the loss increases if the loss of the protection effectiveness is small (0≤*σ*≤*σ_o_*). This implies that a small loss of the protection effectiveness can be compensated by a high optimal prevalence rate of the vaccination program. On the other hand, if the loss is large (*σ_o_*≤*σ*≤1), the optimal rate decreases as the loss of the protection effectiveness increases. This eventuality implies that a large loss of the protection effectiveness is no longer compensated by the high optimal prevalence rate of the vaccination program. Therefore, a low prevalence rate, which does not engender the emergence of a vaccine-resistant strain becomes optimal because the poor vaccine engenders the increase of final size of the epidemic because of the spread of the resistant strain.

We calculate the optimal prevalence rate, which depends on the loss of the protection effectiveness of the vaccination in Fig. 4 (sensitivity analyses are given in Supplementary Information: Text S1, Fig. S6). At the point where the loss of the protection effectiveness is greater than some threshold value *σo*, the optimal prevalence rate changes catastrophically from high prevalence rate to a low prevalence rate. Here
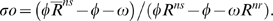



Actually, *σo* = 0.461 in our simulation from Table 1. The optimal prevalence rate is 84.6% when the loss of the protection effectiveness is between 0% and 5.6%. In addition, if the loss rate is between 5.6% and 20.1%, then the optimal prevalence rate increases from 84.6% to 100%. Furthermore, if the loss rate is between 20.1% and 46.1%, then the optimal prevalence rate must always be 100%. Consequently, as long as the loss of the protection effectiveness is small (0%–46.1%), the loss can be compensated by a high optimal prevalence rate of the vaccination program. However, if the loss rate is greater than 46.1%, the loss is no longer compensated by the high prevalence rate of the vaccination program. The optimal prevalence rate changes catastrophically from 100% to 10.2%. Afterward, as the loss rate increases from 46.1% to 100%, the optimal prevalence rate decreases from 10.2% to 4.72% (the low prevalence rate becomes optimal). This is true because the poor vaccine (with a large loss of the protection) engenders the emergence of the vaccine-resistant strain for the high prevalence rate; in addition, the spread of the resistant strain increases the final size of the epidemic. Therefore, the loss of the protection effectiveness strongly impacts also on the optimal prevalence rate.

### Variation of final size of epidemic according to the vaccination program

**Figure 5:**
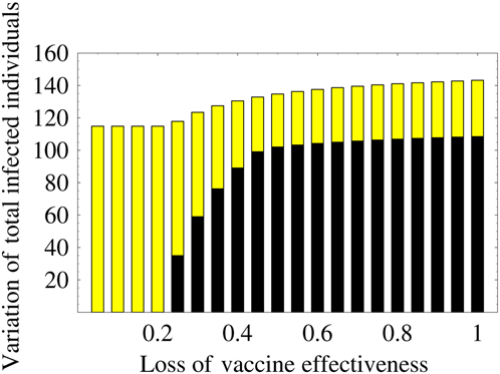
Variation of the final size of the epidemic over the vaccination prevalence: The black bar represents the optimal (smallest) final size of the epidemic. The yellow bar represents the worst (largest) final size of the epidemic over the vaccination prevalence. The variation of the final size depending on the prevalence rate is between black and yellow bars. If the loss of protection effectiveness is small, then the variation is very large. On the other hand, if the loss becomes large, then the variation decreases. Therefore, the final size of the epidemic is strongly affected by the vaccination coverage and the loss of protection effectiveness: a bad vaccination program (far from the optimal prevalence rate) increases the final size and prevents eradication of the disease.

In countries where poultry are mainly backyard scavengers, optimum vaccination coverage might be difficult to achieve [21]. The final size of the epidemic might be increased and the program might fail if the optimal prevalence rate of the vaccination program can not be achieved. However, if we can achieve optimum vaccination coverage, the final size is greatly reduced. The final size of the epidemics can be variable depending on the prevalence rate. Here we calculate the optimal (smallest) and worst (largest) final size of the epidemic over the vaccination prevalence (see Supplementary Information: Text S1) in Fig. 5 (black and yellow bars respectively represent the optimal and worst final size). The variation of the final size is between black and yellow bars shown in Fig. 5 (sensitivity analyses are given in Supplementary Information: Text S1, Fig. S7).

If the loss of protection effectiveness is small, then the variation is very large. The vaccination program can eradicate the disease or reduce the final size of the epidemic to a very small size if we can execute the vaccination program near the optimal prevalence rate. The variation is sensitive for the prevalence rate. Therefore, we must carefully manage the vaccination program to control the disease when the loss is small. However, as the loss of protection effectiveness increases, the variation decreases. In particular, when the loss is medium, the reduction of the variation is remarkable. In addition, the reduction of the variation remains almost unchanged when the loss is large. This implies that the variation becomes insensitive if the loss is high. In this case, even if we can execute the vaccination program near the optimal prevalence rate, the effect of the program is not large. Therefore, although the final size is strongly affected by the vaccination coverage and a non-optimal vaccination program (far from the optimal prevalence rate) increases the final size, in general, good vaccine treatment with small loss of protection effectiveness has a great possibility for disease control. Demonstrably, poor vaccine application has little or no benefit.

### Effects of non-pharmaceutical intervention

Avian influenza vaccination need not be used alone to eradicate the disease: additional non-pharmaceutical intervention is beneficial. Additional interventions must include culling infected animals, strict quarantine, movement controls and increased biosecurity, extensive surveillance [11], [16], [21], [34], [37]. We investigate the effects of some additional non-pharmaceutical intervention measures on the vaccination program. The effects are considered by changing model parameters (1).

In the European Union (EU), regulations for the control of avian influenza strains are imposed by EU council directive 92/40/EEC [34]. Virus output is reduced by the killing and removal of infected poultry flocks (culling). During the H7N7 epidemic in The Netherlands in 2003, this and other approaches were executed. To investigate the effectiveness of the control measures, A. Stegeman *et al.* quantified the transmission characteristics of the H7N7 strain before and after detection of the first outbreak of avian influenza in The Netherlands in 2003 [34]. In Table 1, we present the chosen epidemiological parameters, which are estimated on the H7N7 epidemic before notification of the circulation of the avian influenza (these parameters are not affected by the additional control measures). Here we choose other epidemiological parameters for vaccine-sensitive strain which are estimated by the H7N7 epidemic after the notification in [34] (these parameters are affected by the additional control measures) to evaluate an effect of the non-pharmaceutical intervention on the vaccination program. The estimate of the transmission parameter *ω* decreases considerably from 4.78×10^−4^ day^−1^ individual^−1^ to 1.70×10^−4^ day^−1^ individual^−1^ by the control measures. Furthermore, the estimate of the infectious period 1/(*b*+*my*) is also reduced from 13.8 days to 7.3 days. Therefore, control measures can reduce the basic reproductive number 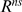 from 6.53 to 1.22 [34]. In addition, we assume, for example, that the relative transmissibility of vaccine-resistant strains is *φ/ω* = 0.7 and that the relative infectious period of vaccine-resistant strain is (*b*+*my*)/(*b*+*mz*) = 1.32 (these values are not strongly influential on our results).

We calculated the threshold values of the loss of protection effectiveness of the vaccination and present them in Table 3 when the vaccination program accompanies non-pharmaceutical intervention. Results show that the non-pharmaceutical intervention markedly reduces the risk of the emergence of the vaccine-resistant strain because *σ* ^*^ changes from 5.6% to 37.2%. In addition, the possibility that the vaccination program eventually eradicates the spread of the disease increases because 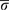 changes from 20.1% to 88.6%. Furthermore, because *σc* changes from 23.6% to 100%, the vaccination program always decreases the final size of the epidemic compared with that before the vaccination program, even if the size increases when both strains co-exist. When the vaccination program accompanies non-pharmaceutical intervention, even if the loss of protection effectiveness is increased considerably by the vaccine-resistant strain, the loss can almost be compensated by the high optimal prevalence rate of the vaccination program: *σo* changes from 46.1% to 96.8%.

**Table 3:** Threshold values of the loss of protection effectiveness of the vaccination.

Loss of protection effectiveness	Threshold values			
	*σ* ^*^	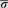	*σc*	*σ_o_*
Before notification of avian influenza	5.6%	20.1%	23.6%	46.1%
After notification of avian influenza	37.2%	88.6%	100%	96.8%

These values are calculated using parameters based on the H7N7 epidemic in The Netherlands in 2003 before and after notification of avian influenza [34].

**Figure 6:**
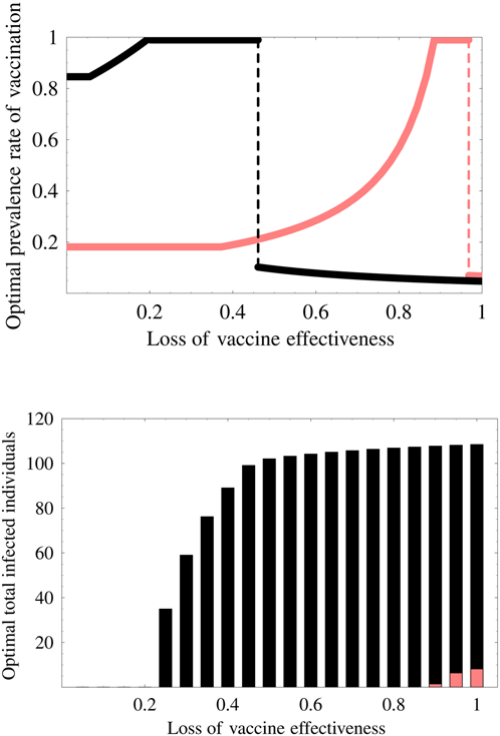
Effects of non-pharmaceutical intervention: The top figure shows the optimal prevalence rate of the vaccination program with (pink curve) or without (black curve) non-pharmaceutical intervention. The non-pharmaceutical intervention readily achieves the optimal prevalence rate and hinders the catastrophic change. The bottom figure shows the optimal final size of the epidemic with (pink bar) or without (black bar) the non-pharmaceutical intervention. The intervention also dramatically reduces the final size of the epidemic.

Figure 6 portrays the optimal prevalence rate of a vaccination program (top figure) and the optimal final size of the epidemic (bottom figure) with (pink curve and bar) or without (black curve and bar) the non-pharmaceutical intervention. The non-pharmaceutical intervention makes it easy to achieve an optimal prevalence rate and to prevent the spread of the disease. Moreover, catastrophic change does not occur until the loss of protection effectiveness becomes very high (top figure in Fig. 6). Furthermore, the optimal final size is also dramatically reduced by the additional intervention (bottom figure in Fig. 6). Even if vaccination without the additional intervention can not prevent the spread of the disease, the vaccination with the intervention can eradicate the disease (for example *σ* = 60%). Therefore, non-pharmaceutical intervention improves weak points of vaccination programs such as the difficult control of optimal vaccination coverage, the small applicability of the program with respect to the loss of protection effectiveness caused by the vaccine-resistant strain, and so on.

### Time-course of the spread of the disease

**Figure 7:**
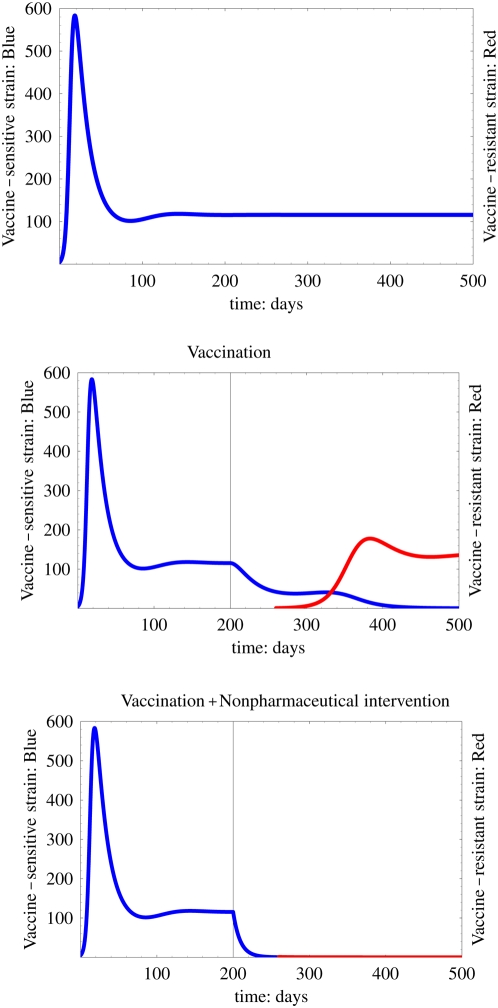
Time-course of the spread of the disease with vaccination and non-pharmaceutical interventions: We calculate epidemic curves with a vaccination program for 500 days. The vaccination program and non-pharmaceutical intervention are started after the vaccine-sensitive strain becomes endemic (around 200 days). We assume that the vaccine-resistant strain occurs after the start of vaccination (around 260 days). The top, middle, and bottom figures respectively depict time courses of infection without the vaccination program, with only the vaccination program, and with both the vaccination program and the non-pharmaceutical intervention. The blue and red curves respectively represent the number of infected individuals with vaccine-sensitive and vaccine-resistant strains. We assume that the prevalence rate of vaccination program is *p* = 0.5, the loss of protection effectiveness is *σ* = 0.8.

Finally, we investigate the time-course of spread of the disease according to vaccination and non-pharmaceutical interventions for 500 days in the presence of a vaccine-resistant strain. The results are presented in Fig. 7. We consider that the vaccination program and non-pharmaceutical interventions are executed after the vaccine-sensitive strain spreads and becomes endemic (around 200 days). Furthermore, the vaccine-resistant strain is assumed to occur in a few individuals after the start of the vaccination program (around 260 days). We assume that the prevalence rate of the vaccination program is *p* = 50%, the loss of protection effectiveness is *σ* = 80%; the other parameters are the same as those used in the descriptions above. These values of *p* and *σ* are not influential on our results (sensitivity analyses are shown in Supplementary Information: Text S1, Fig. S8, S9).

The top figure in Fig. 7 depicts the epidemic curve without the vaccination program. It is apparent that the vaccine-sensitive strain (the blue curve) becomes endemic at around 200 days after a pandemic phase of the disease if we execute no intervention policy. The middle figure portrays the time-course of spread of the disease, assuming the vaccination program alone. A vaccine-resistant strain (the red curve) emerges and spreads widely through the population by replacing the vaccine-sensitive strain. It becomes endemic at around 450 days. This result shows the possibility that the emergence and replacement of the resistant strain can be facilitated by the vaccination program, as in some vaccination programs [19], [21], [22]. We can observe that it takes about several months for the resistant strain to beat the sensitive strain (see the middle figure in Fig. 7). Actually, the replacement time of the resistant strain was reported as several months in the China and Mexico epidemics [19], [21], [22]. The final size of the simulated epidemic is larger than that before (without) the vaccination program because the loss of protection effectiveness *σ* = 80% is greater than 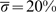 (see Fig. 3). In this case, the vaccination program negatively affects the control of infectious disease. The bottom figure presents the time-course of the spread of the disease with both the vaccination program and non-pharmaceutical interventions. The vaccine-sensitive strain is dramatically reduced and the vaccine-resistant strain hardly spreads in the population; therefore, both strains are eventually controlled at a low level by the interventions. Thus, non-pharmaceutical interventions can help the vaccination program and control the resistant strain to spread in the population.

## Discussion

A serious problem of vaccination strategy is the emergence of vaccine-resistant strains [19], [20], [21], [22]. Even if a resistant strain emerges, a vaccination program must be managed to control the spread of the disease. In the absence of the resistant strain, our mathematical model certainly shows that a large prevalence of the vaccination program might markedly reduce an epidemic curve and the final size of the epidemic. Therefore, we can control infectious diseases as in previous models [30]. However, in the presence of the emergence of a vaccine-resistant strain, the vaccination program can not simply control the spread of the disease. The control of the infectious disease through vaccination becomes more difficult.

The paradoxical result obtained here is that if the virulence of vaccine-resistant strain is less than that of vaccine-sensitive strain, the final size of the epidemic might increase as the prevalence rate of the vaccination program increases (see Fig. 2). A vaccination that is expected to prevent the spread of the disease can instead foster the spread of the disease. Although qualitatively similar results were obtained through more complex models [8], [9], which can be treated analytically only to a slight degree, one of our important results is the clear and simple concept illustrating the value and pitfalls of vaccination programs; the concept can help farmers and administrators to avoid negative effects from paradoxical phenomena.

We investigated how the loss of protection effectiveness impacts a vaccination program's results in the lower virulence case. If the loss of protection effectiveness is between 0 and 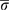, the vaccination program can eventually eradicate the disease, even if a vaccine-resistant strain emerges (see Fig. 3). In particular, if the loss is between 0 and *σ* ^*^, the program prevents even the emergence of the resistant strain. However, when the loss is greater than 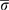, the program no longer prevents the wide spread of the resistant strain in spite of the large prevalence rate of the program. Furthermore, if the loss is between *σc* and 1, the program presents the risk that the final size will become larger than that without the vaccination program. Therefore, in the context of the emergence of the resistant strain, we must carefully execute the program to exercise a positive effect of the vaccine effectively. Additionally, we investigated the optimal prevalence rate of the vaccination program, its final size, and the worst-case final size (see Fig. 4, 5 and Supplementary Information: Text S1). The catastrophic change of the optimal prevalence rate and the variation of the final size depending on the loss of protection effectiveness were confirmed.

From our theoretical analysis, we propose that monitoring the virulence of the resistant strain and investigating the loss resulting from a resistant strain can have important consequences for developing a vaccination strategy. In particular, all thresholds derived herein are only constructed using basic reproductive numbers and transmissibilities that prevail before the vaccination program, which can be estimated using epidemiological data (it is usually almost impossible to estimate basic and invasion reproductive numbers during vaccination programs). Therefore, using our theory, we were able to calculate various risks in the vaccination program using the available data (Table 3) and propose how we might use a poor vaccine, which has a large loss of protection effectiveness, against the resistant strain to maximize the effects of the program (Fig. 4, 5, and 6). For the results reported here, we assumed that the vaccinated birds can perfectly protect the infection from the vaccine-sensitive strain. Although that assumption is not unreasonable [21], in Supplementary Information: Text S1, Fig. S10, S11, we present an investigation of the effect of the loss of protection effectiveness against the vaccine-sensitive strain. Qualitatively similar results were obtained using numerical simulations.

Vaccination is now being used extensively to aid the prevention of emergence or to control the spread of avian influenza [14]. However, if the vaccinations are not used appropriately, prevention and control will be negatively affected by the vaccination program [1], [11], [19], [21], [22]. Actually, when the vaccine-resistant strain emerges, our model predicts various risks in the program. Therefore, to eradicate the infectious disease effectively by vaccination, early detection of the resistant strain, monitoring of its virulence and loss of protection effectiveness of vaccination caused by the resistant strain, and attendance of non-pharmaceutical interventions, in addition to collaboration among farmers, industry, public health authorities, and the government are all required.

## Supporting Information

Figure S1 (0.42 MB EPS)

Figure S2 (0.42 MB EPS)

Figure S3 (0.44 MB EPS)

Figure S4 (0.47 MB EPS)

Figure S5 (0.43 MB EPS)

Figure S6 (1.01 MB EPS)

Figure S7 (1.02 MB EPS)

Figure S8 (1.83 MB EPS)

Figure S9 (1.72 MB EPS)

Figure S10 (0.96 MB EPS)

Figure S11 (1.35 MB EPS)

Text S1 (0.09 MB PDF)
