# Supplementary material for: Paradox of Vaccination: Is Vaccination Really Effective against Avian Flu Epidemics?
Source: PLoS One. 2009 Mar 18;4(3):e4915. doi: 10.1371/journal.pone.0004915 (PMC2657368; doi:10.1371/journal.pone.0004915)
Supplement: Text S1 — (0.09 MB PDF) [file pone.0004915.s012.pdf]

# Supplementary Information for Paradox of vaccination: Is vaccination really effective against avian flu epidemics?

Shingo Iwami, Takafumi Suzuki, Yasuhiro Takeuchi

## 1. Mathematical results

The mathematical properties of model (1) are analyzed completely [4]. The analyses are divisible into three situations related to the vaccination rate as follows.

*(a) Before vaccination program: $p = 0$*

If the prevalence rate of vaccination program is $p = 0$, then model (1) has the following three possible equilibria:

$$E^{n0} = (X^{n0}, 0, 0, 0), \quad \text{where} \quad X^{n0} = \frac{c}{b};$$
$$E^{ns} = (X^{ns}, 0, Y^{ns}, 0), \quad \text{where} \quad X^{ns} = \frac{b + m_y}{\omega}, \quad Y^{ns} = \frac{c - bX^{ns}}{\omega X^{ns}};$$
$$E^{nr} = (X^{nr}, 0, 0, Z^{nr}), \quad \text{where} \quad X^{nr} = \frac{b + m_z}{\phi}, \quad Z^{nr} = \frac{c - bX^{nr}}{\phi X^{nr}}.$$

It also has the following basic and invasion reproductive numbers:

$$R^{ns} = \frac{\omega}{b + m_y} X^{n0}, \quad \bar{R}^{ns} = \frac{\omega}{b + m_y} X^{nr}, \quad R^{nr} = \frac{\phi}{b + m_z} X^{n0}, \quad \bar{R}^{nr} = \frac{\phi}{b + m_z} X^{ns}.$$

Here the left superscript "$n$" means "$p = 0$", the right superscripts "0", "$s$", and "$r$" respectively mean the disease-free equilibrium, vaccine-sensitive strain existing equilibrium, and vaccine-resistant strain existing equilibrium.

The basic reproductive number for vaccine-sensitive (vaccine-resistant) strain $R^{ns}$ ($R^{nr}$) means an expected number of new infectious cases before the spread of any strain among birds [1] and the invasion reproductive numbers for vaccine-sensitive (vaccine-resistant) strain $\bar{R}^{ns}$ ($\bar{R}^{nr}$) means the expected number of new infectious cases after a spread of vaccine-resistant (vaccine-sensitive) strain among birds [4].

We remark that $R^{ns} > R^{nr}$ ($R^{nr} > R^{ns}$) is equivalent to $\bar{R}^{nr} < 1$ ($\bar{R}^{ns} < 1$). The dynamics are determined completely by the basic reproductive numbers $R^{ns}$ and $R^{nr}$ [2, 4].

**Theorem 1.** *(i) If $R^{ns} \leq 1$ and $R^{nr} \leq 1$, then $E^{n0}$ is globally asymptotically stable (GAS), which means that the orbit converges to the equilibrium as $t \to \infty$ for arbitrary initial points.*

*(ii) If $R^{ns} > 1$ and $\bar{R}^{nr} < 1$, then $E^{ns}$ is GAS.*

*(iii) If $R^{nr} > 1$ and $\bar{R}^{ns} < 1$, then $E^{nr}$ is GAS.*

In fact, $\bar{R}^{ns}\bar{R}^{nr} = 1$ and *Theorem 1* includes all cases. The proofs of this theorem are given in [2].

*(b) Complete prevalence of vaccination program:* $p = 1$

If the prevalence rate of vaccination program is $p = 1$, then model (1) has the following two possible equilibria:

$$E^{c0} = (0, V^{c0}, 0, 0), \quad \text{where} \quad V^{c0} = \frac{c}{b};$$
$$E^{cr} = (0, V^{cr}, 0, Z^{cr}), \quad \text{where} \quad V^{cr} = \frac{b + m_z}{\sigma\phi}, \quad Z^{cr} = \frac{c - bV^{cr}}{\sigma\phi V^{cr}};$$

and the following basic reproductive number

$$R^{cr} = \frac{\sigma\phi}{b + m_z} V^{c0}.$$

Here the left superscript "$c$" means "$p = 1$", the right superscript "0" means disease-free equilibrium, and "$r$" means vaccine-resistant strain existing equilibrium.

The dynamical properties are given by the following theorems.

**Theorem 2.** *(i) If $R^{cr} \le 1$, then $E^{c0}$ is GAS.*

*(ii) If $R^{cr} > 1$, then $E^{cr}$ is GAS.*

The proofs of these theorems are presented in [3, 4].

*(c) Incomplete prevalence of vaccination program:* $0 < p < 1$

If the prevalence rate of vaccination program is $0 < p < 1$, then model (1) has the following four possible equilibria:

$$E^{i0} = (X^{i0}, V^{i0}, 0, 0), \quad \text{where} \quad X^{i0} = \frac{(1-p)c}{b}, \quad V^{i0} = \frac{pc}{b};$$
$$E^{is} = (X^{is}, V^{is}, Y^{is}, 0), \quad \text{where} \quad X^{is} = \frac{b + m_y}{\omega}, \quad V^{is} = \frac{pc}{b}, \quad Y^{is} = \frac{(1-p)c - bX^{is}}{\omega X^{is}};$$
$$E^{ir} = (X^{ir}, V^{ir}, 0, Z^{ir}), \quad \text{where} \quad X^{ir} = \frac{(1-p)c}{b + \phi Z^{ir}}, \quad V^{ir} = \frac{pc}{b + \sigma\phi Z^{ir}}.$$

In addition, $Z^{ir}$ is the unique root of the following equations:

$$\frac{\phi(1-p)c}{b + \phi Z} + \frac{\sigma\phi pc}{b + \sigma\phi Z} = b + m_z.$$

$$E^{i+} = (X^{i+}, V^{i+}, Y^{i+}, Z^{i+}), \quad \text{where} \quad X^{i+} = \frac{b + m_y}{\omega}, \quad V^{i+} = \frac{1}{\sigma}\left(\frac{b + m_z}{\phi} - \frac{b + m_y}{\omega}\right),$$
$$Y^{i+} = \frac{1}{\omega}\left\{\frac{(1-p)c - bX^{i+}}{X^{i+}} - \phi Z^{i+}\right\}, \quad Z^{i+} = \frac{pc - bV^{i+}}{\sigma\phi V^{i+}}.$$

The following are basic and invasion reproductive numbers:

$$R^{is} = \frac{\omega}{b+m_y} X^{i0}, \quad \bar{R}^{is} = \frac{\omega}{b+m_y} X^{ir},$$
$$R^{ir} = \frac{\phi}{b+m_z} X^{i0} + \frac{\sigma\phi}{b+m_z} V^{i0}, \quad \bar{R}^{ir} = \frac{\phi}{b+m_z} X^{is} + \frac{\sigma\phi}{b+m_z} V^{is}.$$

Therein, the left superscripts "$i$" means "$0 < p < 1$", the right superscripts "0", "$s$", "$r$", and "+", respectively signify the disease-free equilibrium, vaccine-sensitive strain existing equilibrium, vaccine-resistant strain existing equilibrium, and the both-strains-existing equilibrium. In fact, these basic and invasion reproductive numbers depend on the prevalence rate of vaccination programs.

The dynamical properties are given by the following theorems.

**Theorem 3.** *(i) If $R^{is} \le 1$ and $R^{ir} \le 1$, then $E^{i0}$ is GAS.*

*(ii) If $R^{is} > 1$ and $\bar{R}^{ir} \le 1$, then $E^{is}$ is GAS.*

*(iii) If $R^{ir} > 1$ and $\bar{R}^{is} \le 1$, then $E^{ir}$ is GAS.*

*(iv) If $\bar{R}^{is} > 1$ and $\bar{R}^{ir} > 1$, then $E^{i+}$ is GAS.*

Here, we must note that the relation between these reproductive numbers; $\bar{R}^{is} < 1 < R^{is}$ and $\bar{R}^{ir} < 1 < R^{ir}$ can not hold simultaneously (the relation is provable directly by tedious and complex analysis, but it was clear in *Theorem 3*). Therefore, *Theorem 3* includes all cases. The proofs of this theorem are given in [4].

## 2. Evaluation of the effects of a vaccination program

We investigate how the vaccination affects the total number of infected individuals at each equilibrium (i.e., the final size of the epidemic). We differentiate the total number of infected individuals at $E^{is}$ with respect to a prevalence rate of the vaccination program $p$ as

$$\frac{dY^{is}}{dp} = -\frac{c}{\omega X^{is}} < 0.$$

This implies that increasing the prevalence rate decreases the total number of infected individuals (the vaccination is effective).

The differentiation of the total number at $E^{ir}$ with respect to $p$ is the following:

$$\frac{dZ^{ir}}{dp} = \frac{-bc\phi(1-\sigma)}{\sqrt{\{\sigma\phi^2 c - b\phi(b+m_z)(\sigma+1)\}^2 - 4\sigma\phi^2(b+m_z)b\{b^2+m_z b - c\phi(1-p) - \sigma\phi pc\}}} < 0,$$

$$\frac{d^2Z^{ir}}{dp^2} = \frac{-2\sigma\phi^2(b+m_z)\left(\dfrac{dZ^{ir}}{dp}\right)^2}{\sqrt{\{\sigma\phi^2 c - b\phi(b+m_z)(\sigma+1)\}^2 - 4\sigma\phi^2(b+m_z)b\{b^2+m_z b - c\phi(1-p) - \sigma\phi pc\}}} < 0.$$

The first equation implies that increasing the prevalence rate decreases the total number of infected individuals. The second equation means that the effect of vaccination becomes stronger as $p$ increases.

The differentiation of the numbers of infected individuals with vaccine-sensitive and vaccine-resistant strain at $E^{i+}$ with respect to $p$ are as follows:

$$\frac{dY^{i+}}{dp} = -\frac{c}{\omega}\left(\frac{1}{X^{i+}} + \frac{1}{\sigma V^{i+}}\right) < 0, \quad \frac{dZ^{i+}}{dp} = \frac{c}{\sigma\phi V^{i+}} > 0.$$

Therefore, increasing the prevalence rate decreases the number of infected individuals with the vaccine-sensitive strain but increases the number of infected individuals with the vaccine-resistant strain. Furthermore, differentiation of the total number of infected individuals is given by the following equation:

$$\frac{d(Y^{i+} + Z^{i+})}{dp} = \frac{c\omega(m_y - m_z)}{(b + m_y)\{\omega(b + m_z) - \phi(b + m_y)\}}.$$

Because we assume that $\bar{R}^{nr} < 1$, we have the following relations between the effect of vaccination and the virulence of vaccine-sensitive and vaccine-resistant strain:

$$\frac{d(Y^{i+} + Z^{i+})}{dp} > 0 \Longleftrightarrow m_y > m_z,$$
$$\frac{d(Y^{i+} + Z^{i+})}{dp} = 0 \Longleftrightarrow m_y = m_z,$$
$$\frac{d(Y^{i+} + Z^{i+})}{dp} < 0 \Longleftrightarrow m_y < m_z.$$

These imply that the virulence of each strain plays an important role in the effectiveness of the vaccination program (from the above mathematical analysis, we need not perform a sensitivity analysis of the effect of the vaccination program).

## 3. Impact of loss of protection effectiveness of vaccination

We investigate the impact of the loss of the protection on the change of the final size of the epidemic over the vaccination prevalence. Our basic assumptions are that $R^{ns} > 1$, $R^{nr} > 1$, and that $\bar{R}^{nr} < 1$.

First, we consider the case in which $R^{cr} > 1$. In fact, $R^{is}$ and $\bar{R}^{is}$ become 0 at $p = 1$. Because $R^{cr} > 1$, we can show that $\bar{R}^{ir} > 1$ with $p = 1$. Then, $R^{ns} > R^{nr}$ (that is, $\bar{R}^{nr} < 1$), which implies that $0 < p_a < 1$ (see Fig.1). Here

$$p_a = \frac{1}{\sigma}\left(\frac{1}{R^{nr}} - \frac{1}{R^{ns}}\right)$$

is satisfied with $\bar{R}^{ir}(p_a) = 1$. Actually, $\bar{R}^{is} < 1 < R^{is}$ and $\bar{R}^{ir} < 1 < R^{ir}$ can not hold simultaneously. Therefore, if we can show that $p_a < \bar{p}$, then $\bar{R}^{is} > 1$ for $0 < p < p_a$ (see Fig. 1). Here

$$\bar{p} = 1 - \frac{1}{R^{ns}}$$

is satisfied with $R^{is}(\bar{p}) = 1$. Then, because $\bar{R}^{is}$ is a monotonically decreasing function of $p$ and $\bar{R}^{is}(1) = 0$, we can show that $0 < p_a < p_b < 1$ (see Fig. 1). Here

$$p_b = 1 - \frac{1}{R^{ns}} \left\{ \left(1 - \frac{1}{\sigma}\right)(1 - \bar{R}^{nr}) + R^{nr} \right\}$$

is satisfied with $\bar{R}^{is}(p_b) = 1$. In fact, we can prove that $p_a < \bar{p}$ as

$$\begin{aligned} p_a < \bar{p} &\iff \frac{b}{\sigma c}\left(\frac{b+m_z}{\phi} - \frac{b+m_y}{\omega}\right) < 1 - \frac{b(b+m_y)}{c\omega} \\ &\iff 1 + \frac{b+m_y}{\omega}\frac{\phi}{b+m_z}(\sigma - 1) < \frac{\sigma\phi}{b+m_z}\frac{c}{b}. \end{aligned}$$

Because $0 < \sigma < 1$ and $R^{cr} > 1$, we can show that $p_a < \bar{p}$, which means $0 < p_a < p_b < 1$ (see Fig. 1). Therefore, from *Theorems 1–3*, the stable equilibrium changes

$$E^{ns} \to E^{is} \to E^{i+} \to E^{ir} \to E^{cr}$$

as $p$ increases if $R^{cr} > 1$. In fact, $E^{is}$ ($E^{ns}$) is stable if $0 \le p < p_a$, $E^{i+}$ is stable if $p_a \le p < p_b$, and $E^{ir}$ ($E^{cr}$) is stable if $p_b \le p \le 1$.

Second, we consider cases $R^{cr} < 1$ and $p^* < \bar{p}$. Here,

$$p^* = \frac{1}{1-\sigma}\left(1 - \frac{1}{R^{nr}}\right)$$

is satisfied with $R^{ir}(p^*) = 1$. In this case, we have two possible situations (a) $\bar{R}^{ir} < 1$ for $0 \le p \le 1$ and (b) $\bar{R}^{ir} > 1$ with $p = 1$ (see Fig. 2). In case (a), from *Theorems 1-3*, $E^{is}$ ($E^{ns}$) is stable if $0 \le p < \bar{p}$ and $E^{io}$ ($E^{co}$) is stable if $\bar{p} \le p \le 1$. In case (b), we can show that $\bar{R}^{ir}(p^*) < 1$ as follows.

$$\begin{aligned} \bar{R}^{ir}(p^*) < 1 &\iff 1 + \frac{\sigma}{1-\sigma}\left(\frac{\omega}{b+m_y}\frac{c}{b} - \frac{b+m_z}{\phi}\frac{\omega}{b+m_y}\right) < \frac{b+m_z}{\phi}\frac{\omega}{b+m_y} \\ &\iff 1 + \frac{\sigma}{1-\sigma}(R^{ns} - \bar{R}^{ns}) < \bar{R}^{ns} \\ &\iff 0 < (\bar{R}^{ns} - 1) + \sigma(1 - R^{ns}) \\ &\iff p^* < \bar{p} \end{aligned}$$

Therein, $\bar{R}^{ir}$ is a monotonically increasing function of $p$. Therefore, we can show that $\bar{R}^{ir} < 1$ for $0 < p < p^*$ (see Fig. 2). Therefore, similarly, $E^{is}$ ($E^{ns}$) is stable if

$0 \le p < \bar{p}$ and $E^{io}$ ($E^{co}$) is stable if $\bar{p} \le p \le 1$. Consequently, the stable equilibrium changes

$$E^{ns} \to E^{is} \to E^{i0} \to E^{c0}$$

as $p$ increases if $R^{cr} < 1$ and $p^* < \bar{p}$.

Third, we consider case $R^{cr} < 1$ and $p^* > \bar{p}$. We have the following relations.

$$\begin{aligned}\bar{R}^{ir}(1) > 1 &\iff \frac{\phi}{b+m_z}\frac{b+m_y}{\omega} + \frac{\sigma\phi}{b+m_z}\frac{c}{b} > 1\\ &\iff 1 - \bar{R}^{ns} + \sigma R^{ns} > 0\end{aligned}$$

In fact, $p^* > \bar{p}$ is equivalent to $1 - \bar{R}^{ns} + \sigma R^{ns} > \sigma$. Therefore, we can show that $\bar{R}^{ir} > 1$ with $p = 1$. Furthermore, the following relations hold:

$$\begin{aligned}p_a < p_b \iff &\frac{b}{\sigma c}\left(\frac{b+m_z}{\phi} - \frac{b+m_y}{\omega}\right)\\ &< 1 - \frac{b+m_y}{c\omega}\left\{b(1-\frac{1}{\sigma})\left(1 - \frac{\phi}{b+m_z}\frac{b+m_y}{\omega}\right) + \frac{\phi c}{b+m_z}\right\}\\ \iff &\bar{R}^{ns} - 1 < \sigma R^{ns}(1-\bar{R}^{nr}) - (\sigma-1)(1-\bar{R}^{nr})\\ \iff &0 < (1-\bar{R}^{nr})\{\bar{R}^{nr}(\sigma R^{ns} - \sigma + 1) - 1\}.\end{aligned}$$

In fact, $p^* > \bar{p}$ is equivalent to $\bar{R}^{nr}(\sigma R^{ns} - \sigma + 1) - 1 > 0$. Therefore, because $\bar{R}^{nr} < 1$, we can show that $p_a < p_b$. In addition, we can show that $p_b < \bar{p}$ as follows:

$$\begin{aligned}p_b < \bar{p} \iff &1 - \frac{b(b+m_y)}{c\omega}\left\{\left(1-\frac{1}{\sigma}\right)\left(1 - \frac{\phi}{b+m_z}\frac{b+m_y}{\omega}\right) + \frac{\phi c}{b(b+m_z)}\right\}\\ &< 1 - \frac{b(b+m_y)}{c\omega}\\ \iff &(\sigma-1)\left(\frac{\omega}{b+m_y}\frac{b+m_z}{\phi} - 1\right) + \frac{\sigma c\omega}{b(b+m_y)} > \frac{\sigma\omega}{b+m_y}\frac{b+m_z}{\phi}\\ \iff &(\sigma-1)(\bar{R}^{ns} - 1) + \sigma R^{ns} > \sigma\bar{R}^{ns}\\ \iff &(1-\bar{R}^{ns}) + \sigma(R^{ns} - 1) > 0.\end{aligned}$$

In those expressions, $p^* > \bar{p}$ is equivalent to $(1-\bar{R}^{ns}) + \sigma(R^{ns}-1) > 0$. Therefore, we can obtain $p_b < \bar{p}$. The following order for $p$ holds: $0 < p_a < p_b < \bar{p} < p^* < 1$. Consequently, from *Theorems 1-3*, the stable equilibrium changes

$$E^{ns} \to E^{is} \to E^{i+} \to E^{ir} \to E^{i0} \to E^{c0}$$

as $p$ increases if $R^{cr} < 1$ and $p^* > \bar{p}$. In fact, $E^{is}$ ($E^{ns}$) is stable if $0 \le p < p_a$, $E^{i+}$ is stable if $p_a \le p < p_b$; in addition, $E^{ir}$ is stable if $p_b \le p < p^*$, and $E^{i0}$ ($E^{c0}$) is stable if $p^* \le p \le 1$.

We can summarize the impact of the loss of protection effectiveness of vaccination as follows: Define

$$\sigma^* = \frac{\bar{R}^{ns} - 1}{R^{ns} - 1}, \quad \bar{\sigma} = \frac{1}{R^{nr}},$$

which are satisfied with $0 < \sigma^* < \bar{\sigma} < 1$ (where $\sigma^* < \bar{\sigma}$ is equivalent to $1 < R^{nr}$). The following relations hold for $0 < \sigma < 1$:

$$\begin{aligned} &1 < R^{cr} \Longleftrightarrow \bar{\sigma} < \sigma < 1, \\ &R^{cr} < 1,\ p^* < \bar{p} \Longleftrightarrow 0 < \sigma < \sigma^*, \\ &R^{cr} < 1,\ p^* > \bar{p} \Longleftrightarrow \sigma^* < \sigma < \bar{\sigma}. \end{aligned}$$

Therefore, the change of the total number of infected individuals by vaccination is divisible into the three patterns (see Fig. 4). In addition, because we assume that $m_y > m_z$ in Fig.4, the total number always increases if both strains co-exist. In case $m_y < m_z$, we can also observe these three patterns, although the total number always decreases as $p$ increases. As inferred from results of the mathematical analysis presented above, we need not perform a sensitivity analysis about the change of the total number of infected individuals.

## 4. Vaccination can facilitate spread of disease

We investigate conditions in which the vaccination can help the spread of the disease under $m_y > m_z$. Assume that $\sigma^* < \sigma < 1$. This is true because the vaccination always prevents the spread of the disease if $0 < \sigma < \sigma^*$ (see Fig. 4). Define

$$T_0 = \frac{c\omega - b(b+m_y)}{\omega(b+m_y)}, \quad T_b = \frac{c}{b+m_z} + \frac{(1-\sigma)b(b+m_y)}{\sigma\omega(b+m_z)} - \frac{b}{\sigma\phi},$$

where $T_0$ represents the total number of infected individuals before the vaccination program ($p = 0$) and $T_b$ represents the total number with $p = p_b$ (see Fig. 4). We can evaluate it as follows:

$$\begin{aligned} T_0 < T_b \Longleftrightarrow & \frac{c\omega - b(b+m_y)}{\omega(b+m_y)} < \frac{c}{b+m_z} + \frac{(1-\sigma)b(b+m_y)}{\sigma\omega(b+m_z)} - \frac{b}{\sigma\phi} \\ \Longleftrightarrow & \frac{c\omega}{b(b+m_y)} - 1 < \frac{\omega}{\sigma\phi}\left\{\frac{\sigma c\phi}{b(b+m_z)} + \frac{(1-\sigma)\phi(b+m_y)}{\omega(b+m_z)} - 1\right\} \\ \Longleftrightarrow & \sigma\{\phi(R^{ns} - 1) - \omega(R^{nr} - \bar{R}^{nr})\} < \omega(\bar{R}^{nr} - 1). \end{aligned}$$

We remark that $\phi(R^{ns} - 1) - \omega(R^{nr} - \bar{R}^{nr}) < 0$ is equivalent to $m_y > m_z$. Therefore, we can obtain the following relation:

$$T_0 < T_b \Longleftrightarrow \tilde{\sigma} = \frac{\omega(\bar{R}^{nr} - 1)}{\phi(R^{ns} - 1) - \omega(R^{nr} - \bar{R}^{nr})} < \sigma.$$

In fact, $\sigma^* < \tilde{\sigma}$. Consequently, if $\sigma^* < \sigma < \sigma_c$, then $T_0 > T_b$, where $\sigma_c = \min\{\tilde{\sigma},\ 1\}$. On the other hand, if $\sigma_c < \sigma < 1$, then $T_0 < T_b$. Therefore, when the loss of protection effectiveness is high, the total number becomes larger than that before the vaccination program.

## 5. Difficulty of prediction of a prevalent strain

We show that a strain having a smaller basic reproductive number can beat another strain having the larger one. We assume that $R^{cr} > 1$ ($\bar{\sigma} < \sigma < 1$). If $p_e < p < p_a$, then from *Theorems 1–3*, the vaccine-sensitive strain is shown to be able to beat the vaccine-resistant strain in spite of $R^{ir} > R^{is}$ (see Fig. 5). On the other hand, if $p_b < p < p_e$, then the vaccine-resistant strain can beat the vaccine-sensitive strain in spite of $R^{ir} < R^{is}$ (see Fig. 5). Here

$$p_e = \frac{\bar{R}^{ns} - 1}{\bar{R}^{ns} - 1 + \sigma}$$

is satisfied with $R^{is}(p_e) = R^{ir}(p_e)$. We can also obtain the same results in case $R^{cr} < 1$ ($\sigma^* < \sigma < \bar{\sigma}$).

## 6. Optimal prevalence rate of vaccination program

We investigate an optimal prevalence rate of vaccination program under $m_y > m_z$, which minimizes both the total number of infected individuals and the prevalence rate. If $0 < \sigma < \sigma^*$, then the optimal prevalence rate is $\bar{p}$ (see Fig. 4). If $\sigma^* < \sigma < \bar{\sigma}$, then the optimal prevalence rate is $p^*$ (see Fig. 4). In case $\bar{\sigma} < \sigma < 1$, we can obtain the optimal prevalence rate as follows: Define

$$T_a = \frac{c}{b+m_y} - \frac{b}{\omega} + \frac{b}{\sigma(b+m_y)}\left(\frac{b+m_y}{\omega} - \frac{b+m_z}{\phi}\right), \quad T_1 = \frac{\sigma\phi c - b(b+m_z)}{\sigma\phi(b+m_z)},$$

where $T_a$ represents the total number of infected individuals with $p = p_a$, and $T_1$ represents the total number after vaccination with complete prevalence ($p = 1$). If $T_a < T_1$, then $p = p_a$ is the optimal prevalence rate. On the other hand, if $T_a > T_1$, then $p = 1$ is the optimal prevalence rate. In relation to that point, we offer the following:

$$\begin{aligned} T_a < T_1 &\Longleftrightarrow \frac{c}{b+m_y} - \frac{b}{\omega} + \frac{b}{\sigma(b+m_y)}\left(\frac{b+m_y}{\omega} - \frac{b+m_z}{\phi}\right) < \frac{\sigma\phi c - b(b+m_z)}{\sigma\phi(b+m_z)} \\ &\Longleftrightarrow \sigma\phi\frac{\omega}{b+m_y}\frac{c}{b} - \sigma\phi - \sigma\omega\frac{\phi}{b+m_z}\frac{c}{b} < \phi\frac{\omega(b+m_z)}{\phi(b+m_y)} - \omega - \phi \\ &\Longleftrightarrow \sigma(\phi R^{ns} - \phi - \omega R^{nr}) < \phi\bar{R}^{ns} - \phi - \omega. \end{aligned}$$

Because $\phi(R^{ns}-1)-\omega(R^{nr}-\bar{R}^{nr})<0$ is equivalent to $m_y>m_z$, we can show that $\phi R^{ns}-\phi-\omega R^{nr}<0$. Therefore, we can obtain the following relation:

$$T_a<T_1 \Longleftrightarrow \sigma_o=\frac{\phi \bar{R}^{ns}-\phi-\omega}{\phi R^{ns}-\phi-\omega R^{nr}}<\sigma.$$

Consequently, if $\bar{\sigma}<\sigma<\sigma_o$, then $T_a>T_1$, which also implies that the optimal prevalence rate is $p=1$. On the other hand, if $\sigma_o<\sigma<1$, then $T_a<T_1$, which also implies that the optimal rate is $p=p_a$.

We perform sensitivity analysis to investigate the effect of unestimated parameter change on the optimal prevalence rate shown by the simulation using baseline values (see Table 1). In the top and bottom four figures, we respectively sample the relative mean infectious period of the vaccine-resistant strain $(b+m_y)/(b+m_z)$ from the range of $[1,2]$ ($m_z\in[0.026,0.062]$) and the relative transmissibility of the vaccine-resistant strain $\phi/\omega$ from the range of $[0.4,0.8]$ ($\phi\in[1.91\times10^{-4},3.82\times10^{-4}]$). The other parameters are the same as those presented in Fig. 4 in the main article. From the top four figures, it is apparent that the catastrophic change is apt to occur in the low prevalence rate of the program when $m_z$ is small. Furthermore, from the bottom four figures, when the transmissibility of the vaccine-resistant strain $\phi$ is large, achievement of the optimal prevalence rate becomes difficult. For that reason, increasing the basic reproductive number of vaccine-resistant strain $R^{ir}$ imparts a negative effect on the vaccination program's efficacy.

## 7. Variation of final size of epidemic according to the vaccination program

We investigate a variation of final size of the epidemic by vaccination program depending on the prevalence rate. The variation is between the smallest and largest total number of infected individuals. The smallest total number of infected individuals is given as the following: If $0<\sigma<\bar{\sigma}$, then the optimal total number of infected individuals is 0. If $\bar{\sigma}<\sigma<\sigma_o$, then the optimal total number is $T_1$. Furthermore, if $\sigma_o<\sigma<1$, then $T_a$. On the other hand, the largest number of infected individuals is given as follows: If $0<\sigma<\sigma_c$, then the worst total number of infected individuals is $T_0$. If $\sigma_c<\sigma<1$, then the worst total number is $T_b$.

We perform sensitivity analysis to investigate the effect of unestimated parameter change on the variation of the final size shown using the simulation with baseline values (see Table 1). In the top and bottom four figures, we respectively sample the virulence of the vaccine-resistant strain $m_z$ from the range of $[0.026,0.062]$, the transmissibility of the vaccine-resistant strain $\phi$ from the range of $[1.91\times10^{-4},3.82\times$

$10^{-4}$]. The other parameters are the same as those presented in Fig. 5 in the main article. The variation is more sensitive for $m_z$ than for $\phi$. From the top four figures, it is apparent that the variation widens and the worst total number increases dramatically as $m_z$ decreases. On the other hand, from the bottom four figures, the variation seems to be reduced as $\phi$ increases and the worst total number changes only slightly.

## 8. Time-course of the spread of the disease

Using the parameters given in Table 1 as a default, we varied some parameters to test their effect on the time-course of the spread of the disease shown by numerical simulations in Fig. 7 in the main article.

First, we perform sensitivity analysis about the time-course of the spread of the disease with a vaccination program to change only the unestimated parameters. In the top, upper middle, lower middle, and bottom four figures of Fig. 8, we respectively sample the virulence of the vaccine-resistant strain $m_z$ from the range of $[0.026, 0.062]$, the transmissibility of the vaccine-resistant strain $\phi$ from the range of $[1.91 \times 10^{-4}, 3.82 \times 10^{-4}]$, the prevalence rate of vaccination program $p$ from the range of $[0.4, 0.7]$, and the loss of protection effectiveness of vaccination $\sigma$ from the range of $[0.5, 0.9]$. The other parameters are the same as those presented in Fig. 7 in the main article. The top four figures show that the relative mean infectious period of the vaccine-resistant strain seems to play an important role on the final size of the epidemic. As $m_z$ increases, the final size is reduced. Furthermore, from the lower middle figures, the prevalence rate is shown to have a large effect on the replacement time of the resistant strain. The replacement time becomes shorter as the prevalence rate $p$ increases because the vaccine-sensitive strain dies out rapidly from the high-prevalence vaccination program. However, in almost all figures, the replacement time of the resistant strain seems to be about several months; the final size of the epidemic increases to greater than one before (without) the vaccination program. Therefore, we can conclude that the qualitative behaviors are preserved for variable parameter changes.

Second, similarly, we also perform a sensitive analysis about the time-course of the spread of the disease with vaccination and non-pharmaceutical interventions. In the top, upper middle, lower middle, and bottom four figures of Fig. 9, we respectively sample the virulence of the vaccine-resistant strain $m_z$ from the range of $[0.058, 0.137]$, the transmissibility of the vaccine-resistant strain $\phi$ from the range of $[0.68 \times 10^{-4}, 1.36 \times 10^{-4}]$, the prevalence rate $p$ from the range of $[0.4, 0.7]$, and the loss of protection effectiveness $\sigma$ from the range of $[0.5, 0.9]$. The other parameters

are the same as those presented in Fig. 7 in the main article. The vaccine-sensitive strain is dramatically reduced and the vaccine-resistant strain hardly spread in the population. Therefore, both strains are eventually controlled at a low level by the interventions in almost all figures.

## 9. Incomplete protection against vaccine-sensitive strain

We investigate an effect of incomplete protection against vaccine-sensitive strain. In model (1), we assumed that the vaccinated birds can give perfect protection from infection by the vaccine-sensitive strain. Here we relax that assumption: we assume that the vaccinated individuals can protect the infection from vaccine-sensitive strains at the rate $0 \leq 1-\delta \leq 1$, satisfying $\delta < \sigma$. Therefore, our mathematical model is rewritten as

$$
\begin{aligned}
X' &= (1-p)c - bX - (\omega Y + \phi Z)X, \\
V' &= pc - bV - (\delta\omega Y + \sigma\phi Z)V, \\
Y' &= \omega Y(X + \delta V) - (b + m_y)Y, \\
Z' &= \phi Z(X + \sigma V) - (b + m_z)Z.
\end{aligned}
$$

Using the parameters in Table 1 as default, we varied $\delta$ to test its effects on the final size of epidemics.

First, we investigate the effect of the vaccination program. The parameters are fixed as $\sigma = 0.35$ and $m_z = 0.045$ (lower virulence case) or 0.065 (higher virulence case) as in Fig. 2 in the main article; $\delta$ is sampled from the range of $[0, 0.2]$ [5]. The patterns of the final size are also divisible into two cases that depend strongly on the virulence of the vaccine-resistant strain (see Fig. 10). If $m_y > m_z$ (top figures), then the total number can increase during some prevalence rates, but if $m_y < m_z$ (bottom figures), then the total number always decreases. The two patterns are qualitatively preserved for variable $\delta$, although the effect of the loss of protection effectiveness against a vaccine-sensitive strain delays the emergence of the vaccine-resistant strain.

Second, we investigate the impact of the loss of protection effectiveness against vaccine-sensitive and vaccine-resistant strains. The loss of protection effectiveness against vaccine-resistant strains are fixed as $\sigma = 0.05$, 0.15, and 0.8, similarly to that presented in Fig. 3 in the main article. In addition, $\delta$ is sampled from the range of $[0, 0.2]$. The patterns of the change are also divisible into three cases (see Fig. 11). If $\sigma$ is small (top figures), then the vaccination can control the epidemic without the emergence of a vaccine-resistant strain. If $\sigma$ is medium sized (middle figures), then

the vaccination eventually prevents the spread of the disease. However, if $\sigma$ is large (bottom figures), then the vaccination no longer controls the disease. Although we can also observe that the effect of the loss of protection effectiveness against vaccine-sensitive strain delays the emergence of vaccine-resistant strain, the three patterns are qualitatively preserved for variable $\delta$.

# References


[1] Anderson RM, May RM (1991) Infectious disease of humans: dynamics and control. Oxford University Press.

[2] Bremermann HJ, Thieme HR (1989) A competitive exclusion principle for pathogen virulence. J Math Biol 27: 179-190.

[3] Iwami S, Takeuchi Y, Liu X (2007) Avian-human influenza epidemic model. Math Biosci 207: 1-25.

[4] Iwami S, Takeuchi Y, Liu X, Nakaoka S, A geographical spread of vaccine-resistance in avian influenza epidemics, In Revision.

[5] Peyre M, Fusheng G, Desvaux S, Roger F (2008) Avian influenza vaccines: a practical review in relation to their application in the field with a specifically examine the Asian experience. Epidemiol Infect 14: 1-21.


Figure 1: Basic and invasion reproductive numbers as a function of $p$ with $R^{cr} > 1$: The left panel portrays relations between basic reproductive numbers and the prevalence rate of the vaccination program $p$. The black and red lines respectively depict the basic reproductive number of vaccine-sensitive and the vaccine-resistant strain. The right panel represents relations between invasion reproductive numbers and $p$. The black and red lines respectively signify the invasion reproductive numbers of vaccine-sensitive and vaccine-resistant strains. Actually, BRN and IRN respectively represent the "basic reproductive number" and "invasion reproductive number". Here $\bar{p}$, $p_a$, $p_b$ are satisfied with $R^{is}(\bar{p}) = 1$, $\bar{R}^{ir}(p_a) = 1$, $\bar{R}^{is}(p_b) = 1$, respectively.

Figure 2: Basic and invasion reproductive numbers as a function of $p$ with $R^{cr} < 1$ and $p^* < \bar{p}$: The left panel represents relations between basic reproductive numbers and $p$. The black and red lines respectively signify the basic reproductive number of vaccine-sensitive and vaccine-resistant strain. The right panel represents relations between the invasion reproductive number of vaccine-resistant strain and $p$. ((a) $\bar{R}^{ir} < 1$ for $0 \leq p \leq 1$ and (b) $\bar{R}^{ir} > 1$ with $p = 1$). Here $p^*$ is satisfied with $R^{ir}(p^*) = 1$.

Figure 3: Basic and invasion reproductive numbers as a function of $p$ with $R^{cr} < 1$ and $p^* > \bar{p}$: The left panel portrays relations between basic reproductive numbers and $p$. The black and red lines respectively depict the basic reproductive number of vaccine-sensitive and vaccine-resistant strains. The right panel presents relations between invasion reproductive numbers and $p$. The black and red lines respectively display the invasion reproductive number of vaccine-sensitive and vaccine-resistant strain.

Figure 4: Impact of the loss of protection effectiveness of vaccination on all infected individuals: The change of the total number of infected individuals is classifiable into three cases under $m_y > m_z$: left – $0 < \sigma < \sigma^*$; middle – $\sigma^* < \sigma < \bar{\sigma}$; and right – $\bar{\sigma} < \sigma < 1$. Here $T_0$, $T_a$, $T_b$, and $T_1$ respectively signify the total numbers with $p = 0$, $p = p_a$, $p = p_b$, and $p = 1$.

Figure 5: Basic and invasion reproductive numbers as a function of $p$ with $R^{cr} > 1$ ($\bar{\sigma} < \sigma < 1$). The left panel presents relations between basic reproductive numbers and the prevalence rate of vaccination program $p$. The black and red lines respectively signify the basic reproductive number of vaccine-sensitive and vaccine-resistant strain. The right panel presents relations between invasion reproductive numbers and $p$. The black and red lines respectively depict the invasion reproductive number of vaccine-sensitive and vaccine-resistant strain. Here $p_e$ is satisfied with $R^{is}(p_e) = R^{ir}(p_e)$.

Figure 6: Sensitivity analysis of the relative mean infectious period of the vaccine-resistant strain and the relative transmissibility of the vaccine-resistant strain for the optimal prevalence rate of the vaccination program: We respectively sample $m_z$ from the range of $[0.026, 0.062]$ and $\phi$ from the range of $[1.91 \times 10^{-4}, 3.82 \times 10^{-4}]$.

Figure 7: Sensitivity analysis of the relative mean infectious period of the vaccine-resistant strain and the relative transmissibility of the vaccine-resistant strain for the variation of the final size of the epidemic. We respectively sample $m_z$ from the range of $[0.026, 0.062]$ and $\phi$ from the range of $[1.91 \times 10^{-4}, 3.82 \times 10^{-4}]$.

Figure 8: Sensitivity analysis of the relative mean infectious period of the vaccine-resistant strain, the relative transmissibility of the vaccine-resistant strain, the prevalence rate of vaccination program, and the loss of protection effectiveness of vaccination for the time-course of spread of the disease with vaccination program: We respectively sample $m_z$ from the range of $[0.026, 0.062]$, $\phi$ from the range of $[1.91 \times 10^{-4}, 3.82 \times 10^{-4}]$, $p$ from the range of $[0.4, 0.7]$, $\sigma$ from the range of $[0.5, 0.9]$. The qualitative behaviors are preserved for variable parameter change.

Figure 9: Sensitivity analysis of the relative mean infectious period of the vaccine-resistant strain, the relative transmissibility of the vaccine-resistant strain, the prevalence rate of vaccination program, and the loss of protection effectiveness of vaccination about the time-course of spread of the disease with vaccination and non-pharmaceutical interventions: We respectively sample $m_z$ from the range of $[0.058, 0.137]$, $\phi$ from the range of $[0.68 \times 10^{-4}, 1.36 \times 10^{-4}]$, $p$ from the range of $[0.4, 0.7]$, $\sigma$ from the range of $[0.5, 0.9]$. The qualitative behaviors are preserved for variable parameter change.

Figure 10: Sensitivity analysis of loss of protection effectiveness against vaccine-sensitive strain $\delta$ for the effect of the vaccination program: We assume that $\sigma = 0.35$, $m_z = 0.045$ (lower virulence case) or 0.065 (higher virulence case), and $\delta$ is sampled from the range of $[0, 0.2]$. The top and bottom four panels respectively represent the final size of epidemics related with the prevalence rate of vaccination in the case of lower and higher virulence of vaccine-resistant strains.

Figure 11: Sensitivity analysis of loss of protection effectiveness against vaccine-sensitive strain $\delta$: We investigate the impact of the loss of the protection effectiveness of the vaccination $\sigma$ and $\delta$ on the change of final size of the epidemic. The top, middle, and bottom four figures respectively represent cases $\sigma = 0.05$, 0.15, and 0.8. We assume that $\delta$ is sampled from the range of $[0, 0.2]$.
